# A coarse‐to‐fine framework combining deep learning and Monte Carlo for BNCT patient position optimization toward inverse planning

**DOI:** 10.1002/mp.70642

**Published:** 2026-08-20

**Authors:** Yoonho Na, Chang‐min Lee, Kyuri Kim, Hyungjoo Cho, Jimin Lee, Sung‐Joon Ye

**Affiliations:** ^1^ Department of Applied Bioengineering Seoul National University Seoul Republic of Korea; ^2^ T‐ROH Inc. Seoul Republic of Korea; ^3^ Atherosoft Inc. Seoul Republic of Korea; ^4^ Department of Nuclear Engineering Ulsan National Institute of Science and Technology Ulsan Republic of Korea; ^5^ Research Institute for Convergence Science Seoul National University Seoul Republic of Korea; ^6^ Advanced Institute of Convergence Technology Seoul National University Suwon Republic of Korea

**Keywords:** Bayesian optimization, BNCT, deep learning, inverse planning, Monte Carlo, patient positioning

## Abstract

**Background:**

Boron Neutron Capture Therapy (BNCT) utilizes high linear energy transfer (LET) charged particles from the ^10^B(n,α)^7^Li reaction to selectively destroy tumor cells. Unlike conventional radiation therapy, BNCT facilities rely on fixed beam ports, making patient positioning critical in treatment planning. Current treatment planning, however, depends on manual forward planning using computationally intensive Monte Carlo (MC) simulations.

**Purpose:**

We propose an automated patient position optimization framework for BNCT to address these limitations. This method overcomes the computational bottleneck of MC simulations by integrating a rapid deep learning (DL) dose prediction model with a high‐fidelity GPU‐accelerated MC engine, enabling efficient inverse planning.

**Methods:**

We developed a hybrid coarse‐to‐fine optimization workflow driven by the Trust Region Bayesian Optimization (TuRBO) algorithm. In the coarse stage, a 3D U‐Net architecture rapidly identifies promising patient positions. In the fine stage, the solution is refined within a restricted parameter bound using the GPU‐accelerated MC engine. We evaluated the framework on the GLIS‐RT open dataset (229 glioblastoma patients, split into train, validation, and test sets at a 70:20:10 ratio).

**Results:**

Optimizing the patient position with TuRBO improved target dose and homogeneity over a non‐optimized baseline, reaching equivalent plan quality about 5.65× faster than an exhaustive grid search. In the coarse stage, the DL dose predictor ran about 37× faster than the high‐fidelity MC. The coarse‐to‐fine workflow then matched high‐fidelity MC plan quality while cutting optimization time by a factor of 2.7, outperforming optimization driven by the DL predictor alone. Two‐port plans required no change to the method, where the added geometric freedom raised the mean target dose from 59.96% to 74.91% and lowered the homogeneity index (HI) from 1.226 to 0.647.

**Conclusions:**

We successfully developed a Bayesian optimization (BO) framework for BNCT patient positioning. The proposed coarse‐to‐fine approach effectively balances computational speed with accuracy, offering a practical way toward automated inverse planning in BNCT.

## 1 INTRODUCTION

Treating radioresistant and infiltrative tumors is a major hurdle in radiation oncology, particularly where conventional photon‐based external beam radiation therapy (EBRT) offers limited local control. Boron Neutron Capture Therapy (BNCT) addresses this limitation by utilizing tumor‐selective boron‐10 compound to deliver high linear energy transfer (LET) radiation at the cellular scale.^1^ The BNCT relies on the ^10^B(n,α)^7^Li reaction, which is initiated when thermal neutrons are captured by boron‐10 nuclei accumulated within tumor cells. This reaction yields an alpha particle and a lithium nucleus with high LET values of approximately 150 keV/µm and 175 keV/µm, respectively.^2^, ^3^ Because the range of these particles is short in tissue (5‐9 µm), the energy deposition is confined almost exclusively to boron‐containing cells, sparing surrounding normal tissue.^4^ This microscopic selectivity, coupled with a relative biological effectiveness (RBE) of 1.5–3.0, makes BNCT particularly attractive for malignancies such as glioblastoma (GBM) and recurrent head and neck cancers.^5^

Recent advances in accelerator‐based BNCT (AB‐BNCT) systems have significantly expanded access to neutron irradiation, demonstrating encouraging clinical outcomes for recurrent and treatment‐refractory diseases.^6^, ^7^, ^8^, ^9^ Despite these successes, the geometric limitations of current facilities introduce significant challenges for treatment planning. Unlike EBRT systems equipped with rotatable gantries and tables, most BNCT facilities are restricted to fixed beam ports.^10^, ^11^ As a result, the flexibility of selecting beam angles is replaced with the necessity of optimizing the patient's position and orientation relative to a stationary neutron field.^12^ Consequently, patient positioning becomes a critical determinant of planning dose distribution. Setup deviations of just 1–3 cm laterally can reduce the tumor dose by more than 10%, while axial shifts may result in dose reductions of 5–15%.^13^, ^14^ Furthermore, for deep‐seated targets, neutron attenuation and scattering flatten the depth‐dose profile, heightening the risk of underdosing the tumor or overdosing organs at risk (OARs).^15^, ^16^

Current BNCT treatment planning relies on manual forward planning, a process that is both iterative and labor‐intensive.^17^ Planners are required to select candidate positions, execute computationally demanding Monte Carlo (MC) dose calculations, and subsequently evaluate Dose‐Volume Histograms (DVHs).^18^ Since a single high‐fidelity MC simulation can take several hours, an exhaustive exploration of the geometric positioning space is clinically infeasible.^19^ We address these limitations by treating BNCT patient positioning as a high‐dimensional black‐box optimization problem. The main difficulty lies in balancing the need to maximize tumor dose against sparing OARs, while dealing with the massive computational cost of the MC dose engine.^20^ Because optimal positioning depends heavily on individual anatomy and specific boron uptake, we need an optimization strategy that is extremely sample‐efficient.^21^, ^22^

Bayesian Optimization (BO) is well‐suited for this type of problem where gradients are missing and every function evaluation is expensive.^23^, ^24^ BO uses a probabilistic surrogate model, a Gaussian Process, to approximate the value of the objective function and quantify the uncertainty based on the evaluation points; this allows the BO framework to guide the search efficiently using acquisition functions. Similar methods have already proven useful in medical physics tasks that involve complex spaces and stochastic simulations.^25^

In this study, we propose a BO‐based framework for the automated selection of optimal patient positions in BNCT treatment. Our results demonstrate that near‐optimal patient positions can be identified with a limited number of high‐fidelity dose calculations, offering a practical pathway toward automated inverse planning in BNCT.

## 2 MATERIALS AND METHODS

### 2.1 Dataset and preprocessing

GLIS‐RT^26^ open dataset was utilized in this work, comprising 229 glioblastoma patients with CTs and structure sets. The evaluated OARs included the eyes, brainstem, normal tissue, and bone. The eyes and brainstem are conventional CNS radiation OARs and are commonly reported in BNCT studies of high‐grade gliomas.^27^ Under the Studsvik clinical convention used here, the whole normal brain dose sets the dose‐limiting constraint.^28^, ^29^ Bone is included because it still receives dose in the neutron field, from thermal neutron capture reactions on hydrogen and nitrogen.^30^ The CT couch was automatically removed from all CT images using the TotalSegmentator^31^ model. To train the DL‐based dose prediction network, a synthetic dataset was generated by simulating 50 random two‐port beam configurations per patient using RT^2^. ^32^ All spatial data were resampled to a resolution of 128 × 128 × 64 voxels, and intensity values were normalized to [−1, 1]. Using fixed random seeds, the dataset was split at the patient level into train, validation, and test sets with a ratio of 70:20:10, respectively.

### 2.2 Optimization workflow

**FIGURE 1:**
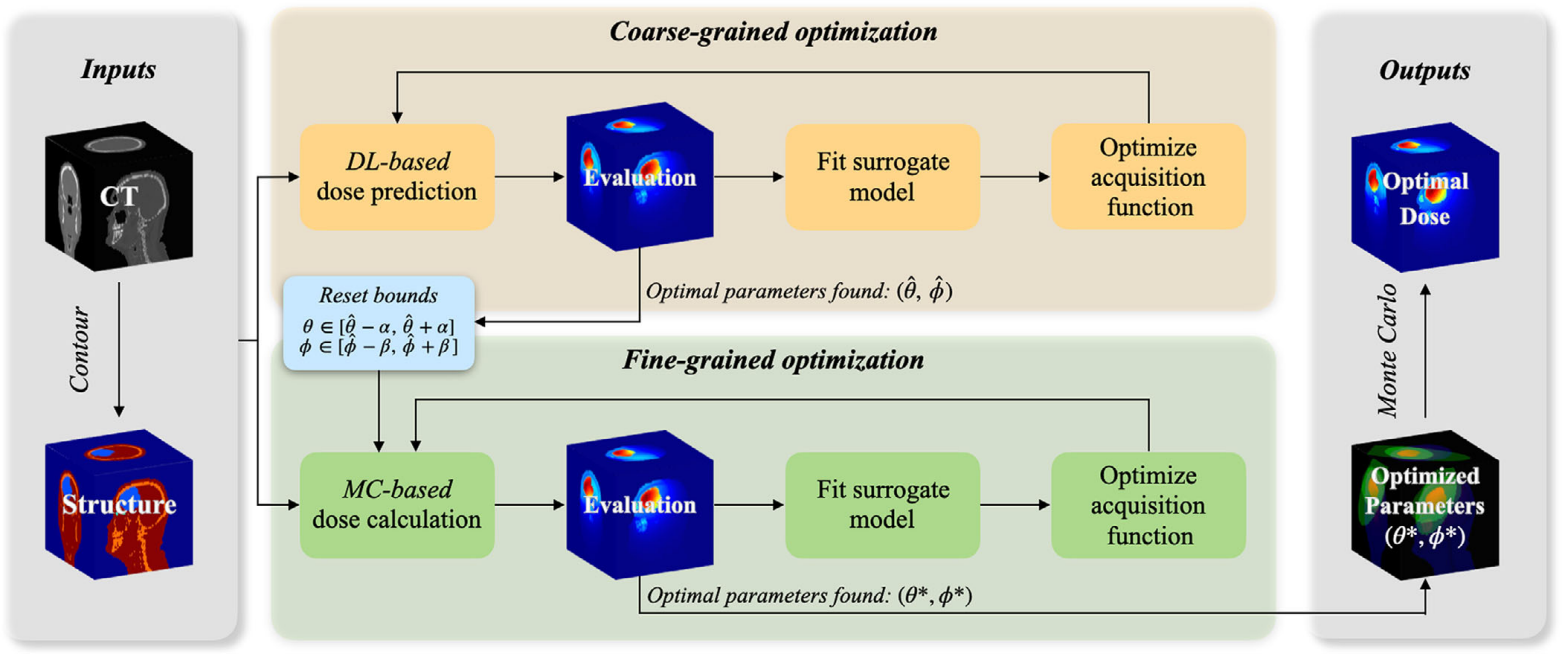
Overview of the coarse‐to‐fine optimization workflow. The proposed optimization workflow begins with a coarse‐grained optimization using a deep learning‐based dose predictor to rapidly identify a promising candidate region. This is followed by a fine‐grained optimization using Monte Carlo simulations within a restricted search space centered on the solution found in the coarse‐grained optimization.

We developed a coarse‐to‐fine optimization framework for BNCT patient positioning, as illustrated in Figure 1. The process consists of two sequential BO loops. First, a *coarse‐grained* stage explores the global search space using a high‐speed DL dose prediction model. This allows for rapid identification of promising anatomical orientations without the computational burden of full MC simulations. Once a candidate region is identified, the workflow transitions to a *fine‐grained* stage. Here, the search is restricted to a local parameter bound centered on the coarse solution. High‐fidelity dose calculations are performed using the GPU‐accelerated MC code RT2 to refine the final beam parameters. Both stages employ Trust Region Bayesian Optimization (TuRBO)^33^ to efficiently navigate the search space.

### 2.3 Dose calculation methods

Our framework uses two dose calculation engines, the GPU‐accelerated Monte Carlo code RT^2^ and a deep learning‐based dose predictor, each used at a different stage of the workflow, to balance speed and accuracy.

#### 2.3.1 GPU‐accelerated Monte Carlo

The fine‐grained optimization and final dose verification rely on a GPU‐accelerated MC, RT^2^, which simulates neutron and secondary particle transport through the voxelized patient geometry to compute the dose. Because each MC evaluation is accurate but computationally expensive, RT^2^ is used only in the fine‐grained optimization, after the search has already been restricted to a small region.

The MC reference and reduced‐history runs were configured with 10^9^ and 10^8^ histories, respectively. For voxels receiving at least 50% of the maximum dose, the mean per‐voxel statistical uncertainty was 0.7% (range, 0.5%–1.1%) at 10^9^ histories and 1.9% (range, 1.5%–2.2%) at 10^8^ histories across the test patients. The 10^8^ history runs are labeled coarse MC in this sense, since their per‐voxel noise is several times that of the reference. Coarsening the dose‐tally grid is another standard MC acceleration strategy, but it gives no meaningful speedup on RT^2^ because the runtime is dominated by particle transport rather than by dose scoring.

We modeled a cylindrical beam with a fixed radius of 6 cm. To reduce the dimensionality of the search space, the beam port position was constrained to a spherical surface with a fixed radius of 15 cm, rather than using Cartesian coordinates. Furthermore, the beam direction vector was constrained to always point toward the target's center of mass. Consequently, the optimization parameter space was reduced to a single polar angle pair (θ,ϕ) for single‐port configurations, and to two pairs with a relative beam weight ω for two‐port configurations.

**FIGURE 2:**
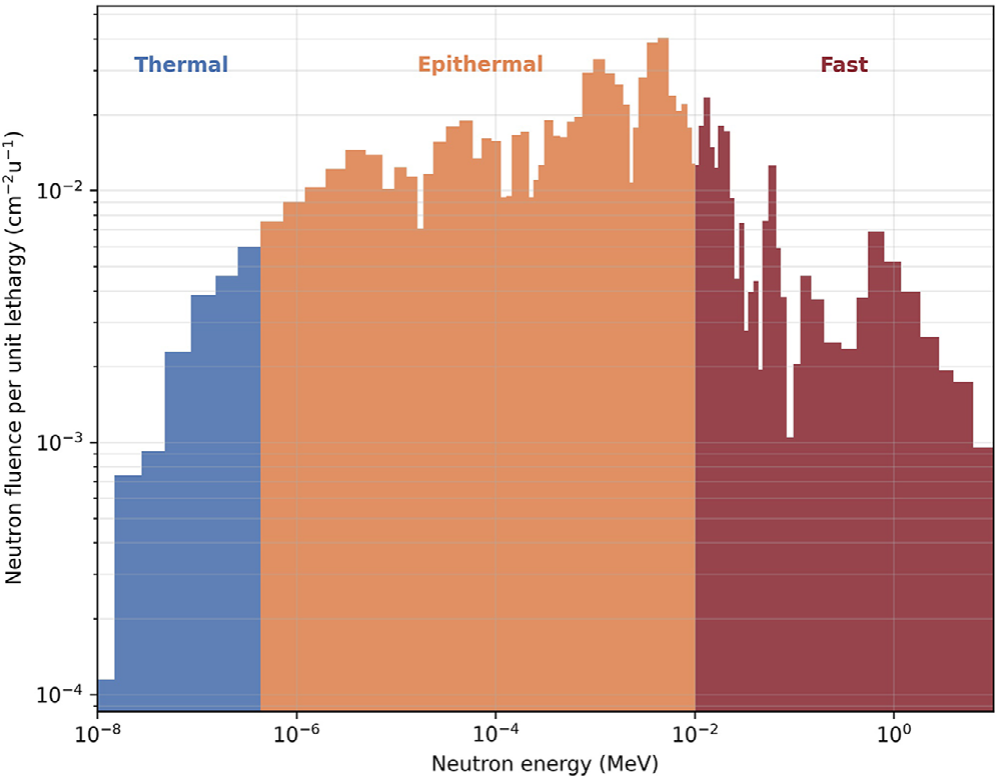
Neutron energy spectrum used as the source configuration in the Monte Carlo simulations. The thermal, epithermal, and fast energy regions are indicated.

Neutron and photon beam spectra were adopted from our previous work.^34^ Figure 2 shows the adopted neutron spectrum. Boron‐10 concentrations were fixed at 60 ppm for the target volume and 18 ppm for normal tissue. All dose values are reported as biological doses (Gy‐Eq), calculated as:

(1) $$D=W_{B}\;\cdot D_{B}+W_{p}\cdot D_{p}+W_{N}\cdot D_{N}+D_{\gamma }$$

where $D_{B},\;D_{p},\;D_{N},$ and $D_{\gamma }$ denote the physical doses from boron, protons, nitrogen, and gamma rays, respectively. The corresponding weighting factors were set as $W_{B}$ = 3.8 (tumor) and 1.4 (normal tissue) for p‐boronophenylalanine (BPA), $W_{p}$ = 3.2, and $W_{N}$ = 3.0, consistent with standard BNCT dosimetry. The boron concentrations and weighting factors used in this study were adopted from previous studies.^35^, ^36^

#### 2.3.2 Deep learning‐based dose prediction

For the coarse‐grained optimization stage, we utilized a 3D U‐Net^37^ architecture to predict 3D dose distributions directly from patient anatomy and beam parameters. The model takes the input of CT images, structure masks, and beam configuration, and outputs four separate dose channels for the boron, proton, nitrogen, and gamma components in Equation (1). The RBE‐weighted total dose used in the objective is then reconstructed by recombining these channels with their weighting factors. Because the boron concentration and weighting factors enter only at this recombination step, and not during training, changing them does not require retraining the model.

The encoder has three downsampling stages with output channel counts of 64, 128, 256, and 512, followed by three middle blocks at the lowest spatial resolution and three upsampling stages linked to the encoder by skip connections. The beam parameters (θ, φ, and the relative beam weight ω for two‐port configurations) are projected to a 256‐dimensional embedding and injected into every block, conditioning the predicted dose on the patient position. The input is a 7‐channel volume comprising the CT, five structure masks (target, eyes, brainstem, tissue, and bone), and a beam mask, and the output is the corresponding 4‐channel dose volume. The model was trained for 500 epochs with the AdamW optimizer (learning rate 1×10^−4^, weight decay 1×10^−3^, batch size 2).

The voxel‐wise MAE within each structure was computed as $\mathrm{MAE}\;=\left(1/N\right)\;\Sigma_{i}\left|D_{\mathrm{pred},i}-D_{\mathrm{MC},i}\right|$, in Gy‐Eq, and the spatial agreement against the 10^9^ history MC reference was evaluated by 3D gamma analysis in the target volume at the 3%/3 mm and 2%/2 mm criteria.

#### 2.3.3 Optimization algorithm

Patient positioning is a non‐convex, black‐box optimization problem. The objective can only be evaluated by running a dose calculation and each evaluation is computationally expensive. BO is well suited to such problems, but conventional BO tends to struggle in high‐dimensional spaces due to over‐exploration. To use limited evaluation budget more efficiently, we employed TuRBO, an established BO variant that confines the search to an adaptive local region of the search space.

TuRBO maintains a trust region centered on the current best solution and restricts the BO search space to that region. The trust region expands after several consecutive evaluations that improve on the current best solution, and shrinks after several consecutive evaluations that do not. This ensures an efficient balance between exploration and exploitation, allowing the algorithm to rapidly converge to the global optimum while evaluating a minimal number of computationally expensive objective functions.

The optimization loop ends under either of two conditions: the trust region size shrinks below a minimum trust region length, indicating local convergence, or the process reaches a predefined maximum iteration limit. We set the minimum trust region length to 0.5^10^(≈9.77×10^−4^) for the coarse‐grained optimization and 0.05 for the fine‐grained optimization, and capped the fine stage at 100 iterations. In angular terms, this coarse‐grained stage threshold corresponds to a trust region narrower than about 0.1° in θ and 0.4° in φ at convergence. The coarse stage therefore narrows the search to a sub‐degree neighborhood before the fine stage begins. The coarse stage was not iteration‐capped, since DL evaluations are inexpensive enough to let it run to convergence.

#### 2.3.4 Objective function and metrics

Equations (2) through (5) define the objective function and the plan quality metric used in this study. The objective function is formulated to penalize excessive OAR exposure and target dose heterogeneity while rewarding target coverage:

(2) $$f\;\left(x\right)=\alpha \cdot T\left(x\right)-\beta \cdot O\left(x\right)\;$$

with the target term

(3) $$T\;\left(x\right)=w_{1}\;\cdot D_{mean}^{target}+w_{2}\cdot D_{98\%}^{target}-w_{3}\cdot \left(\lambda \cdot HI_{target}\right)$$

and the OAR term

(4) $$O\;\left(x\right)=\frac{1}{N_{OAR}}\;\sum_{i}^{N_{OAR}}\left(w_{4,\;i}\cdot D_{mean}^{OAR_{i}}\left(x\right)+w_{5,\;i}\cdot D_{max}^{OAR_{i}}\left(x\right)\right)$$

Here x denotes the patient positioning parameters, and α and β balance the target and OAR contributions. Within the target term, $w_{1}$, $w_{2}$, and $w_{3}$ are relative weights on the mean target dose ($D_{mean}^{target}$), the near‐minimum target dose ($D_{98\%}^{target}$), and the homogeneity index (HI). Because HI is roughly two orders of magnitude smaller than the percentage dose metrics, it is multiplied by a fixed scale factor λ so that it contributes on a comparable numerical magnitude. Within the OAR term, $w_{4,i}$ and $w_{5,i}$ are relative weights on the mean and maximum OAR dose. Notably, the penalty weights $w_{4,i}$ and $w_{5,i}$ are indexed for representing ith organ, allowing clinical planners to assign structural priorities based on individual OAR thresholds. In this implementation, the weighting parameters were fixed at α= 0.75, β= 0.25, $w_{1}$= 0.25, $w_{2}$= 0.5, $w_{3}$= 0.25, $w_{4}$= 0.5, $w_{5}$= 0.5, and λ= 50. For simplicity, the organ‐specific weights $w_{4}$ and $w_{5}$ were set uniformly across all structures. In clinical practice, these per‐organ weights should follow the dose tolerance of each structure. The brainstem is a serial organ with low tolerance to high‐dose regions, so it would take the largest weight on $D_{max}^{OAR_{i}}$, whereas bone tolerates higher doses and would take a smaller weight. The framework allows this per‐organ adjustment directly through the weight values, with no change to the method.

Plan quality was assessed using dose‐volume histograms (DVHs) and the homogeneity index (HI), defined as:

(5) $$HI=\frac{D_{2\%}-D_{98\%}\;}{D_{50\%}}$$

where $D_{x\%}$ is the dose received by *x*% of the target volume. Lower HI values indicate a more homogeneous target dose distribution.

#### 2.3.5 Experimental design

To validate our proposed framework, we designed four comparative experiments. First, we evaluated the impact of beam optimization and configuration by comparing a shortest path baseline (minimizing geometric depth) against single‐port and two‐port optimized configurations, thereby quantifying the dosimetric benefits of optimization and multi‐beam flexibility. Second, to justify the selection of TuRBO, we compared it against Random Search, Grid Search, and standard BO. For this comparison, we restricted the problem to a single‐port configuration to keep the Grid Search computationally feasible. Third, we demonstrated the advantage of using the DL‐based dose prediction method during the coarse‐grained optimization, compared with coarse MC simulation using a smaller number of histories. Finally, we validated our workflow by comparing it against DL‐only and MC‐only baselines, demonstrating the synergy of combining fast approximation with high‐fidelity refinement.

All doses are reported in Gy‐Eq under the convention that the maximum normal tissue dose is normalized to 15 Gy‐Eq, following the protocol adopted at Studsvik Medical AB, which prescribes a peak normal tissue dose of 15 Gy‐Eq in a 1 cm^3^ volume.^29^ For consistency with the voxel‐wise evaluation used throughout this study, we apply the same 15 Gy‐Eq limit to the single‐voxel maximum.

## 3 RESULTS AND DISCUSSION

### 3.1 Validation of the deep learning‐based dose prediction

**FIGURE 3:**
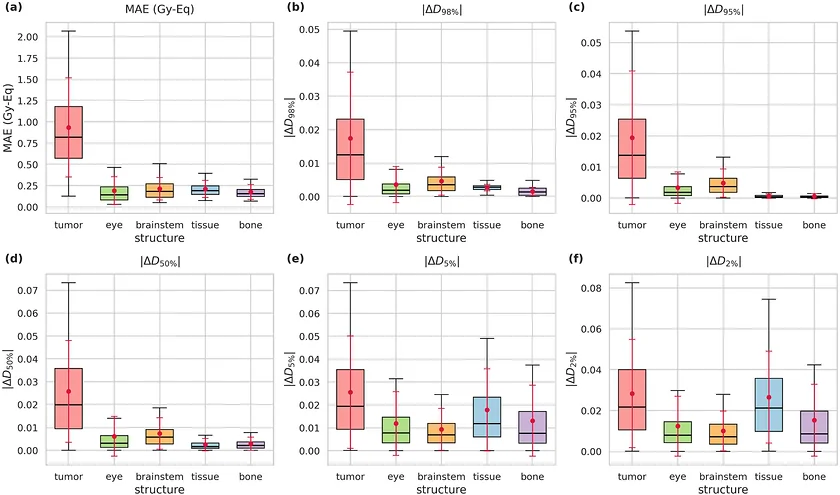
Distribution of mean absolute error (MAE) of the deep learning‐based dose prediction model across the test dataset: (a) voxel‐wise MAE within each anatomical structures, and (b)‐(f) absolute deviations in selected DVH metrics ($|\Delta D_{98\%}\left|,\;\right|\Delta D_{95\%}\left|,\;\right|\Delta D_{50\%}\left|,\;\right|\Delta D_{5\%}|,$ and $|\Delta D_{2\%}|$, respectively). Each box plot depicts the median and interquartile range, and the red markers indicate the mean ± standard deviation.

Before using the DL model in the optimization workflow, we evaluated its accuracy on the held‐out test set against the 10^9^ history MC reference, using two metrics: the voxel‐wise mean absolute error (MAE) within each structure and the gamma passing rate within the target volume. Table 1 summarizes the MAE results. The mean MAE was 0.93 ± 0.58 Gy‐Eq within the target and below 0.22 Gy‐Eq across all OARs. Figure 3 shows the per‐patient distribution of MAE and of the absolute errors on selected DVH metrics. Target errors were larger than OAR errors, which follows from the steeper dose gradients and higher absolute doses in the target. Averaged across the 10 test patients, the target‐volume gamma passing rate was 89.0 ± 10.2% at 3%/3 mm and 77.8 ± 13.9% at the stricter 2%/2 mm. These values show substantial but imperfect voxel‐level agreement, in line with a coarse‐stage surrogate trained under an MSE loss. In our framework the DL model serves to localize a promising region of the search space at low cost, not to produce clinically deployable doses. Any residual bias is removed by the subsequent MC refinement in the fine stage.

**TABLE 1:** Mean absolute error (MAE) of the deep learning‐based dose prediction model for the target and organs at risk, evaluated on the test dataset.

	$|\Delta D_{98\%}|$ (%)	$|\Delta D_{95\%}|$ (%)	$|\Delta D_{50\%}|$ (%)	$|\Delta D_{5\%}|$ (%)	$|\Delta D_{2\%}|$ (%)	MAE (Gy‐Eq)
Target	1.741 ± 1.978	1.941 ± 2.149	2.577 ± 2.224	2.555 ± 2.455	2.834 ± 2.653	0.934 ± 0.582
Eye	0.360 ± 0.539	0.334 ± 0.503	0.612 ± 0.867	1.188 ± 1.390	1.235 ± 1.465	0.192 ± 0.163
Brainstem	0.461 ± 0.417	0.484 ± 0.454	0.739 ± 0.685	0.935 ± 0.913	1.000 ± 0.970	0.214 ± 0.131
Tissue	0.269 ± 0.095	0.064 ± 0.074	0.249 ± 0.272	1.782 ± 1.793	2.657 ± 2.250	0.212 ± 0.100
Bone	0.154 ± 0.120	0.064 ± 0.086	0.290 ± 0.288	1.310 ± 1.551	1.527 ± 1.762	0.177 ± 0.087

*Note*: Data are presented as mean ± standard deviation.

### 3.2 Impact of beam optimization and configuration

We first quantified the benefits of optimization by comparing three configurations across 10 test patients: a non‐optimized shortest path plan, a TuRBO optimized single‐port plan and a TuRBO optimized two‐port plan. The relative impact of the optimization algorithm and the additional beam port were evaluated by comparing the single‐port optimized plan against the shortest path baseline and the two‐port configuration, respectively. Table 2 summarizes the dosimetric outcomes, reported as the mean ± standard deviation across the patients.

**TABLE 2:** Dosimetric comparison of the shortest path, single‐port optimized, and two‐port optimized beam configurations.

Metric	Shortest path	1 port opt	2 port opt
$D_{2\%}\left(\%\right)↑$	95.20±1.62	95.30±2.15	95.05±1.82
$D_{50\%}\left(\%\right)↑$	59.50±10.82	61.50±11.52	76.95±5.79
$D_{98\%}\left(\%\right)↑$	25.70±10.39	27.05±11.14	45.95±8.99
$D_{mean}^{target}\left(\%\right)↑$	59.96±9.19	61.46±9.75	74.91±5.72
HI↓	1.226±0.360	1.171±0.367	0.647±0.146

*Note*: The arrows ↑ and ↓ indicate that a higher or lower value is preferable, respectively.

Compared to the shortest path plan, TuRBO‐optimized single‐port plan yielded a modest increase in mean target dose ($D_{mean}^{target}$) from 59.96% to 61.46% and a reduction in HI from 1.226 to 1.171. The shortest path plan is a reasonable geometric starting point, but it ignores patient anatomy. Optimization adapts the beam orientation to each patient's target and OAR shape, which is what produces the improvement in HI and mean dose reported above.

**FIGURE 4:**
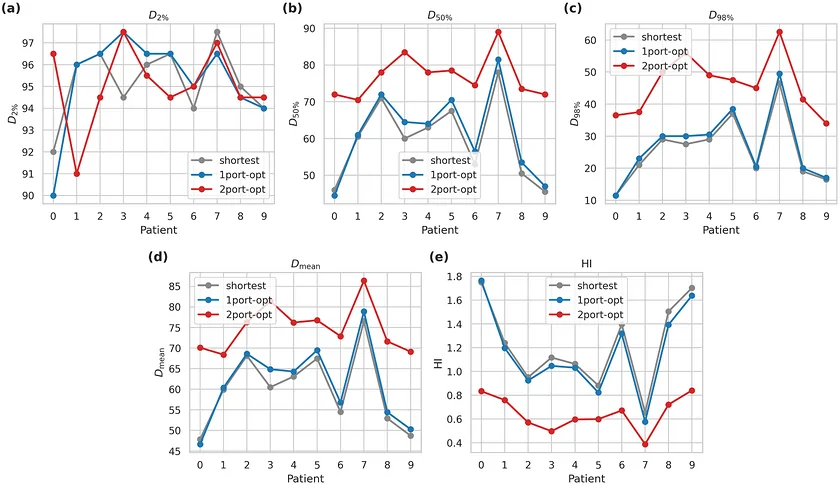
Per‐patient plan quality comparison across test patients for three planning strategies: Shortest path, single‐port optimized, and two‐port optimized. Panels show the clinical target volume metrics for 10 patients: (a) near‐maximum dose ($D_{2\%}$), (b) median dose ($D_{50\%}$), (c) near‐minimum dose ($D_{98\%}$), (d) mean dose ($D_{mean}$), and (e) homogeneity index (HI). Higher values indicate superior target coverage in (a)‐(d), whereas a lower value indicates a more uniform dose distribution in (e).

**FIGURE 5:**
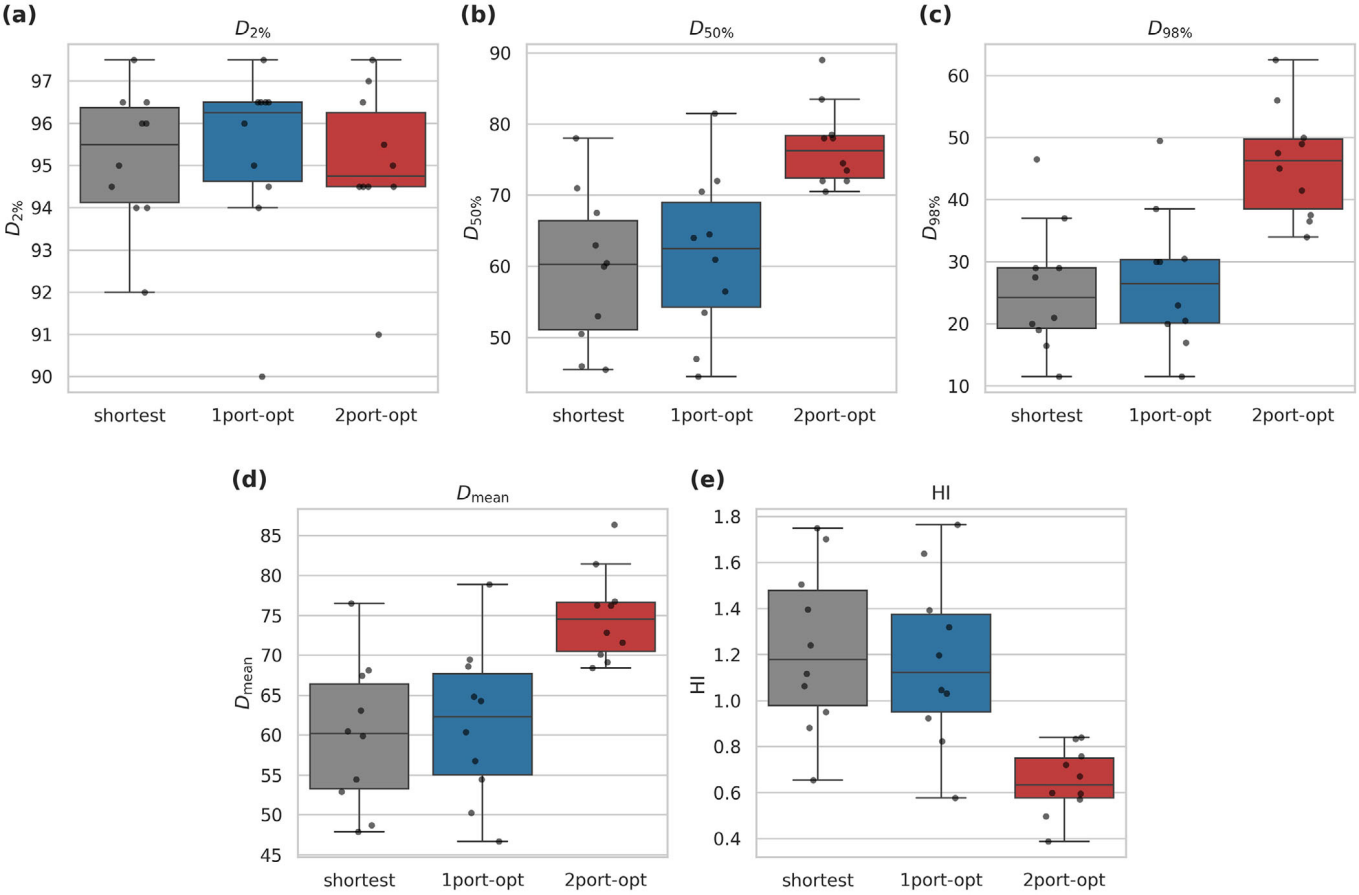
Distributions of plan‐quality metrics across the 10 test patients for the three planning strategies (shortest path, single‐port optimized, and two‐port optimized): (a) near‐maximum dose ($D_{2\%}$), (b) median dose ($D_{50\%}$), (c) near‐minimum dose ($D_{98\%}$), (d) mean dose ($D_{mean}$), and (e) homogeneity index (HI).

**FIGURE 6:**
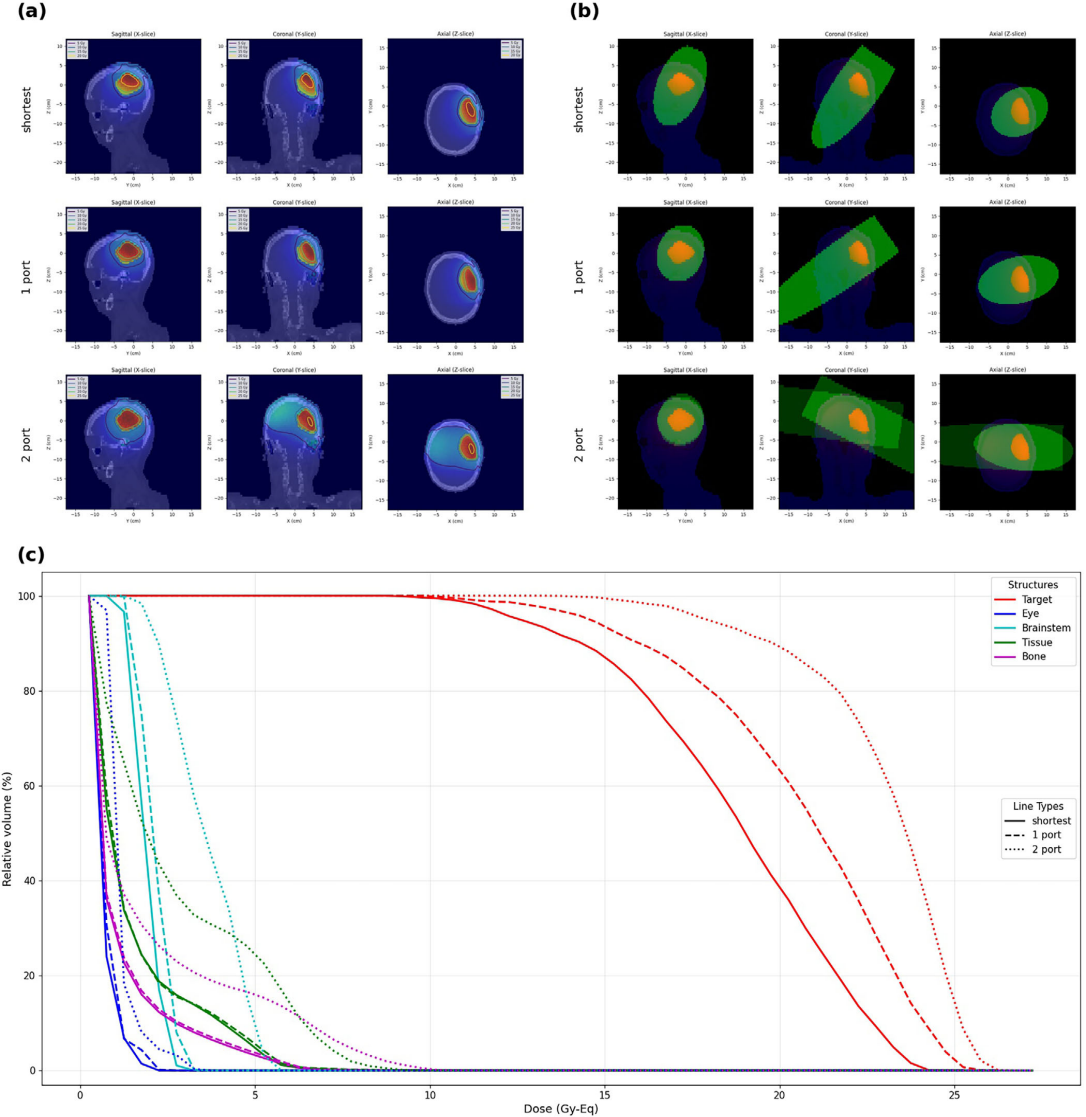
Comparison of beam configurations for a representative patient. (a) Dose distributions for the shortest path, single‐port optimized, and two‐port optimized plans. (b) Beam path projections. (c) Dose‐volume histograms (DVHs) showing the improved target coverage achieved with optimization.

The two‐port results show that the same framework handles multi‐port plans without modification. With a second port, plans for the same patients reached a higher mean target dose and a lower HI, in line with the added geometric freedom of a second beam direction. This gain comes from the multi‐port geometry itself, not from the optimization framework, and we report it only to show that the method extends to multi‐port planning. Figures 4 and 5 show these results per patient and as distributions, and Figure 6 shows the dose distributions for a representative case.

### 3.3 Efficiency of search strategies

The coarse‐to‐fine framework requires a sample efficient optimizer to identify high‐quality treatment plans with minimal dose evaluations, as each objective function evaluation requires an expensive dose calculation. In this study, we adopted TuRBO, an established trust‐region Bayesian optimization algorithm, and this section justifies that choice by benchmarking it against three alternatives (random search, grid search, and standard BO). The comparison was conducted on a single‐port planning configuration for a representative test patient. All evaluations were computed with the same dose engine, RT^2^ at 10^9^ histories. Note that all angles reported in this study are expressed in degrees. All experiments were run on the same workstation under identical settings, with no batched or multi‐process parallelization across candidates, to ensure a fair runtime comparison; the hardware comprised an Intel Core i9‐10900X CPU @ 3.70 GHz (10 cores, 20 threads) and an NVIDIA GeForce RTX 4090 GPU (24 GB VRAM).

Grid search exhaustively sampled a uniform 19 × 73 grid (5° spacing in θ and ϕ), resulting in 1387 evaluations. Random search evaluated 300 uniformly sampled parameters. Standard BO utilized a Gaussian process with a Matérn 5/2 kernel and an upper confidence bound (UCB) acquisition function, initialized with 50 random samples followed by 250 iterations. TuRBO was initialized with 25 random samples and then selected subsequent candidates using the Thompson sampling acquisition function. The run terminated automatically once the trust region length fell below a defined minimum threshold, which occurred after 233 evaluations.

As shown in Table 3, all four strategies converged to similar dosimetric quality ($D_{mean}^{target}$ ≈ 59.5%, HI ≈ 1.22), indicating that the global optimum was successfully identified by each method. However, computational efficiency varied drastically. Grid search was the most expensive, requiring 53 322 s for 1387 evaluations, whereas random search and standard BO each used 300 evaluations (11 489 and 12 390 s, respectively). TuRBO achieved equivalent plan quality with only 9443 s for 233 evaluations. TuRBO was approximately 5.65× faster than grid search, 1.22× faster than random search, 1.31× faster than standard BO. This efficiency stems from TuRBO's local trust region approach, which prevents over‐exploration of unpromising areas in high‐dimensional spaces. We also note that the hyperparameters in TuRBO were not precisely tuned for this specific problem. These results justify TuRBO as the core solver for our framework, particularly for time‐constrained clinical workflows.

**TABLE 3:** Comparison of the four search strategies for a single‐port optimization on a representative test patient. All evaluations used the Monte Carlo dose engine with 109 histories.

		Random	Grid	Standard BO	TuRBO
Beam	θ	33.6844	35	36.0434	34.8274
	ϕ	133.4312	145	143.1211	143.5258
$D_{mean}^{target}\left(\%\right)↑$		59.5854	59.4619	59.1546	59.7374
HI↓		1.2416	1.2166	1.2185	1.225
Number of evaluations ↓		300	1387	300	233
Time (sec) ↓		11489	53322	12390	9443

*Note*: The arrows ↑ and ↓ indicate that a higher or lower value is preferable, respectively.

### 3.4 Comparative evaluation of coarse‐stage dose calculation

All experiments in this subsection were conducted under the two‐port configuration. The performance of the DL dose prediction model in the coarse‐grained optimization stage was benchmarked against a low‐fidelity, reduced‐history coarse MC baseline (10^8^ histories). A high‐fidelity MC calculation (10^9^ histories) served as the reference standard. Note that the DL dose predictor had itself been trained on 10^9^ history MC dose distributions.

**FIGURE 7:**
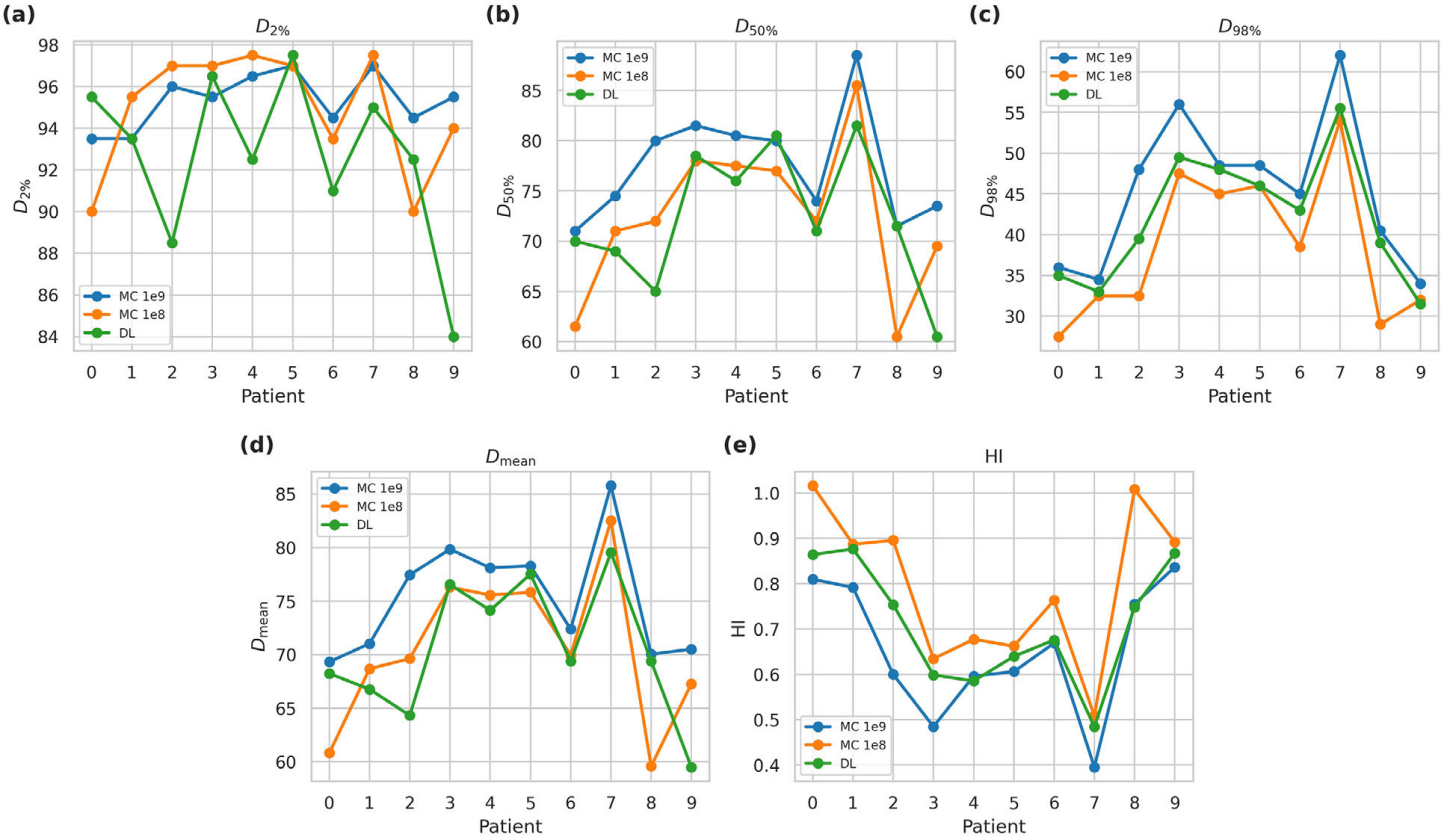
Per‐patient plan quality comparison across the 10 test patients for three dose calculation strategies: high‐fidelity Monte Carlo (MC) simulation with 109 histories, coarse MC simulation with 108 histories, and deep learning‐based dose prediction. Panels show the clinical target volume metrics for patients 0–9: (a) near‐maximum dose ($D_{2\%}$), (b) median dose ($D_{50\%}$), (c) near‐minimum dose ($D_{98\%}$), (d) mean dose ($D_{mean}$), and (e) homogeneity index (HI). Higher values indicate superior target coverage in (a)‐(d), whereas a lower value indicates a more uniform dose distribution in (e).

**FIGURE 8:**
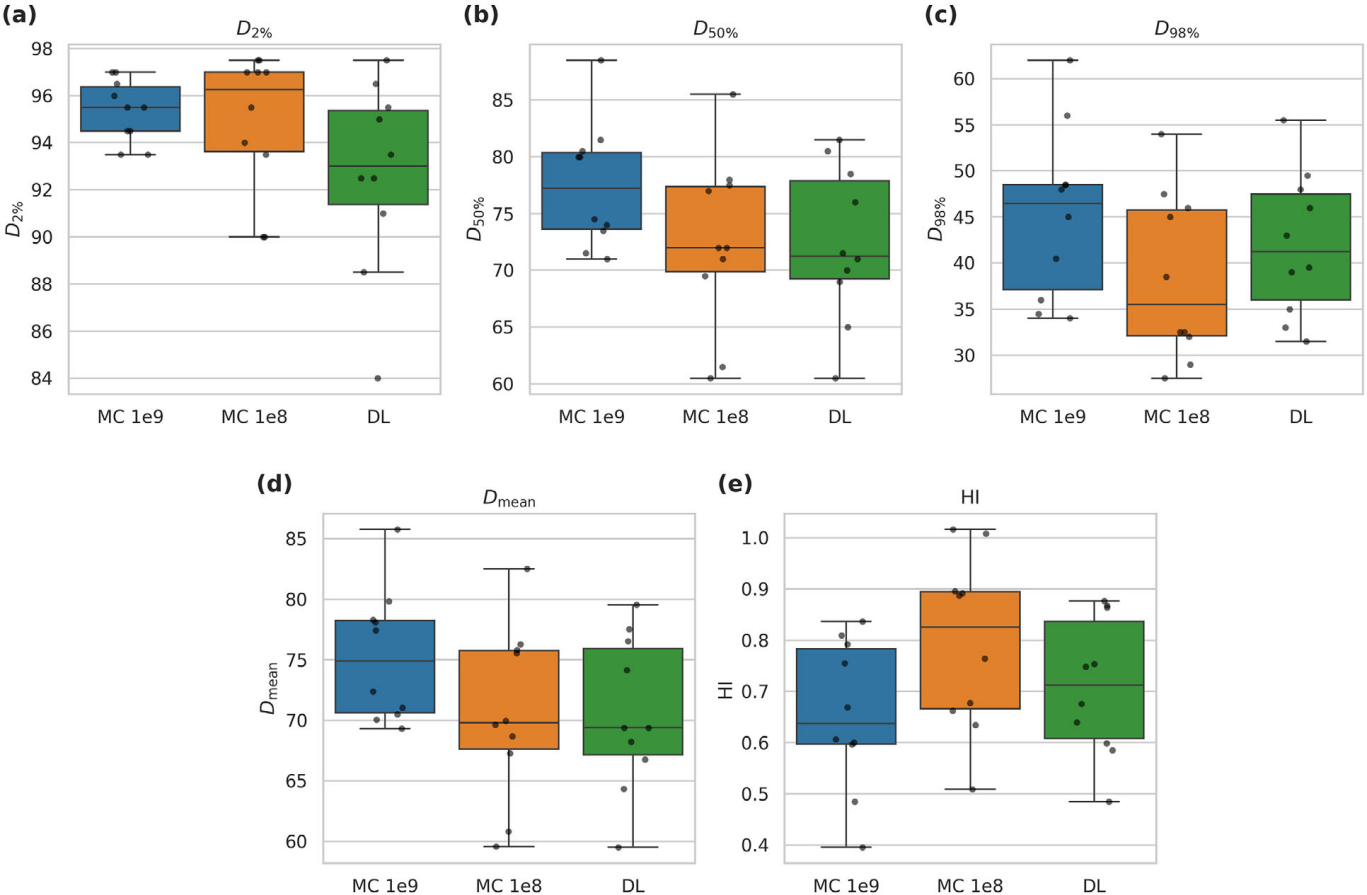
Distributions of plan‐quality metrics across the 10 test patients for the three dose calculation strategies (high‐fidelity MC with 109 histories, coarse MC with 108 histories, and deep learning‐based dose prediction): (a) near‐maximum dose ($D_{2\%}$), (b) median dose ($D_{50\%}$), (c) near‐minimum dose ($D_{98\%}$), (d) mean dose ($D_{mean}$), and (e) homogeneity index (HI).

As shown in Figures 7 and 8, the DL‐based and coarse MC‐based optimizations produced plans of similar overall quality, each falling short of the 10^9^ history reference as expected for an approximate coarse‐stage engine. Quantified as the MAE from the reference across the 10 test patients, the two dose engines were comparable on $D_{50\%}$ and $D_{mean}$ (6%–7% for each), with metric‐specific differences: DL had the smaller error on HI (8.9% versus 21.4%) and $D_{98\%}$ (7.3% versus 15.1%), whereas the coarse MC had the smaller error on $D_{2\%}$. The DL‐based optimization gave a better HI than the coarse MC‐based optimization in all 10 patients and a better $D_{98\%}$ in 8 of the 10, while the two performed comparably on $D_{50\%}$ and $D_{mean}$.

**FIGURE 9:**
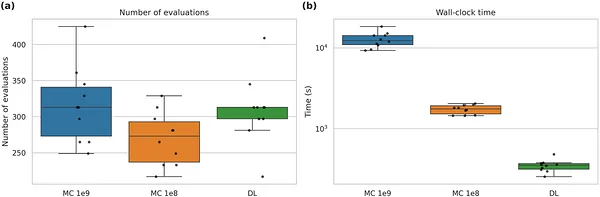
Optimization efficiency across the 10 test patients for the three dose calculation strategies. (a) Total number of evaluations and (b) total wall‐clock time per patient.

The major difference was computational cost (Figure 9). The mean optimization time per patient was 346 s for the DL‐based loop, 1730 s for the coarse MC‐based loop, and 12 764 s for the reference MC‐based loop. Because the three engines required a comparable number of evaluations (≈270‐320), this gap reflects the per‐evaluation cost. The DL‐based dose predictor was therefore approximately 5 × faster than the coarse MC and 37 × faster than the reference MC, while producing plans of comparable overall quality to the coarse MC.

These results also clarify why a DL‐based dose predictor is preferable to a coarse MC calculation for the coarse‐grained stage, regardless of how the coarse MC is configured. Conventional strategies for accelerating MC calculations reduce computational cost by introducing approximations into the dose estimates, which manifest either as increased stochastic variance from fewer histories or as volume‐averaging errors from a coarser calculation grid. A DL predictor instead provides a deterministic, reproducible dose estimate, with no stochastic noise and a fixed bias, at an essentially constant evaluation cost. For the coarse‐grained optimization stage, where the goal is only to localize a promising region quickly, this combination of speed and reproducibility is the crucial advantage. Any bias of the DL prediction is subsequently removed in the fine‐grained stage, where the high‐fidelity MC engine refines the plan within a restricted search space.

### 3.5 Effectiveness of the coarse‐to‐fine workflow

All experiments in this subsection were conducted under the two‐port configuration. We evaluated the proposed coarse‐to‐fine optimization workflow against two single‐loop baselines across the 10 test patients: A DL‐only optimization, driven entirely by the DL dose predictor, and an MC‐only optimization, driven entirely by the high‐fidelity 10^9^ history MC engine. In this experiment, the optimizer (TuRBO) was identical for all three.

**FIGURE 10:**
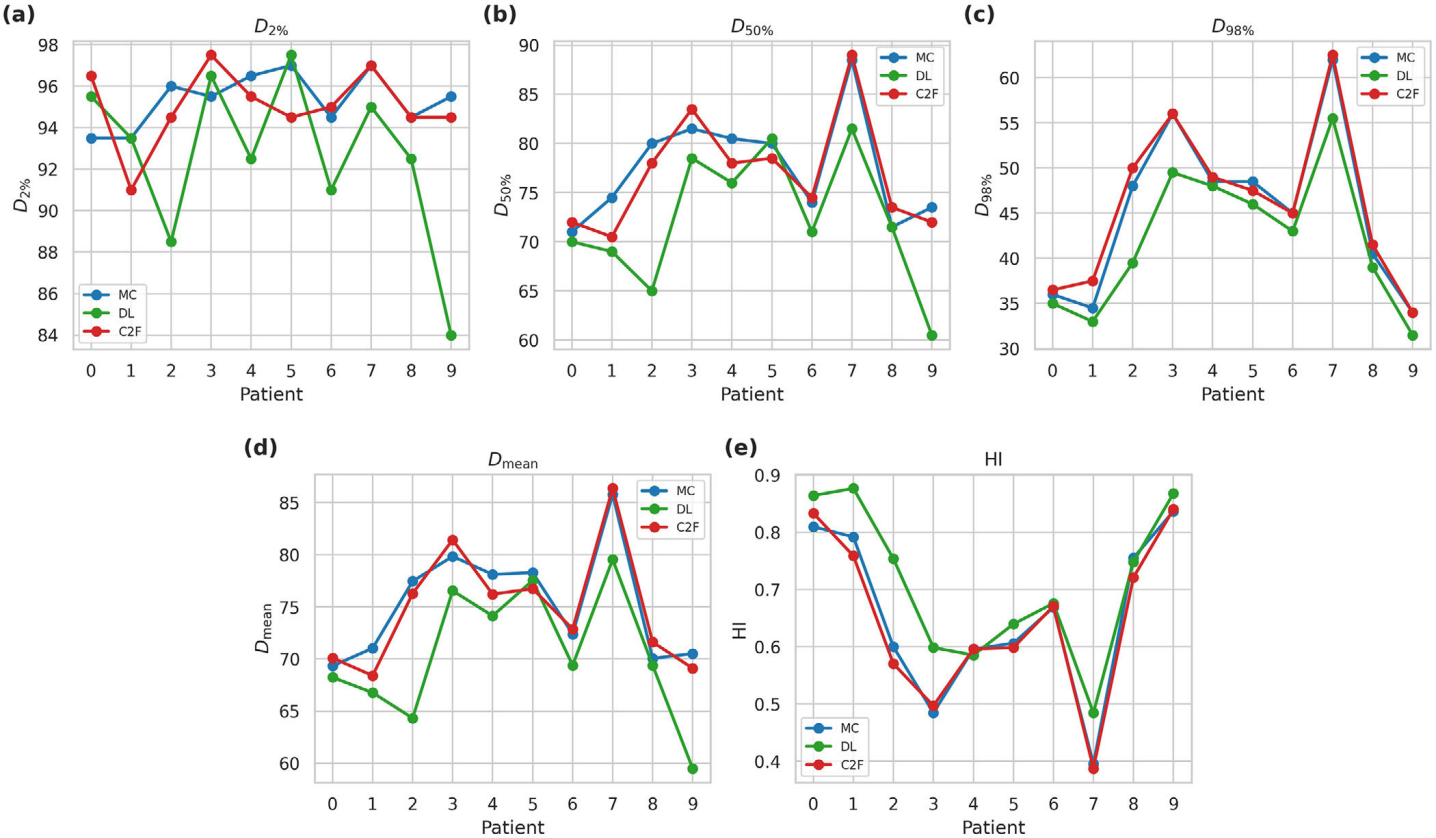
Per‐patient plan quality comparison across the 10 test patients for three optimization strategies: a single‐loop optimization using Monte Carlo (MC), a single‐loop optimization using deep learning‐based dose prediction (DL), and the coarse‐to‐fine optimization (C2F). Panels show the clinical target volume metrics for patients 0–9: (a) near‐maximum dose ($D_{2\%}$), (b) median dose ($D_{50\%}$), (c) near‐minimum dose ($D_{98\%}$), (d) mean dose ($D_{mean}$), and (e) homogeneity index (HI). Higher values indicate superior target coverage in (a)‐(d), whereas a lower value indicates a more uniform dose distribution in (e).

**FIGURE 11:**
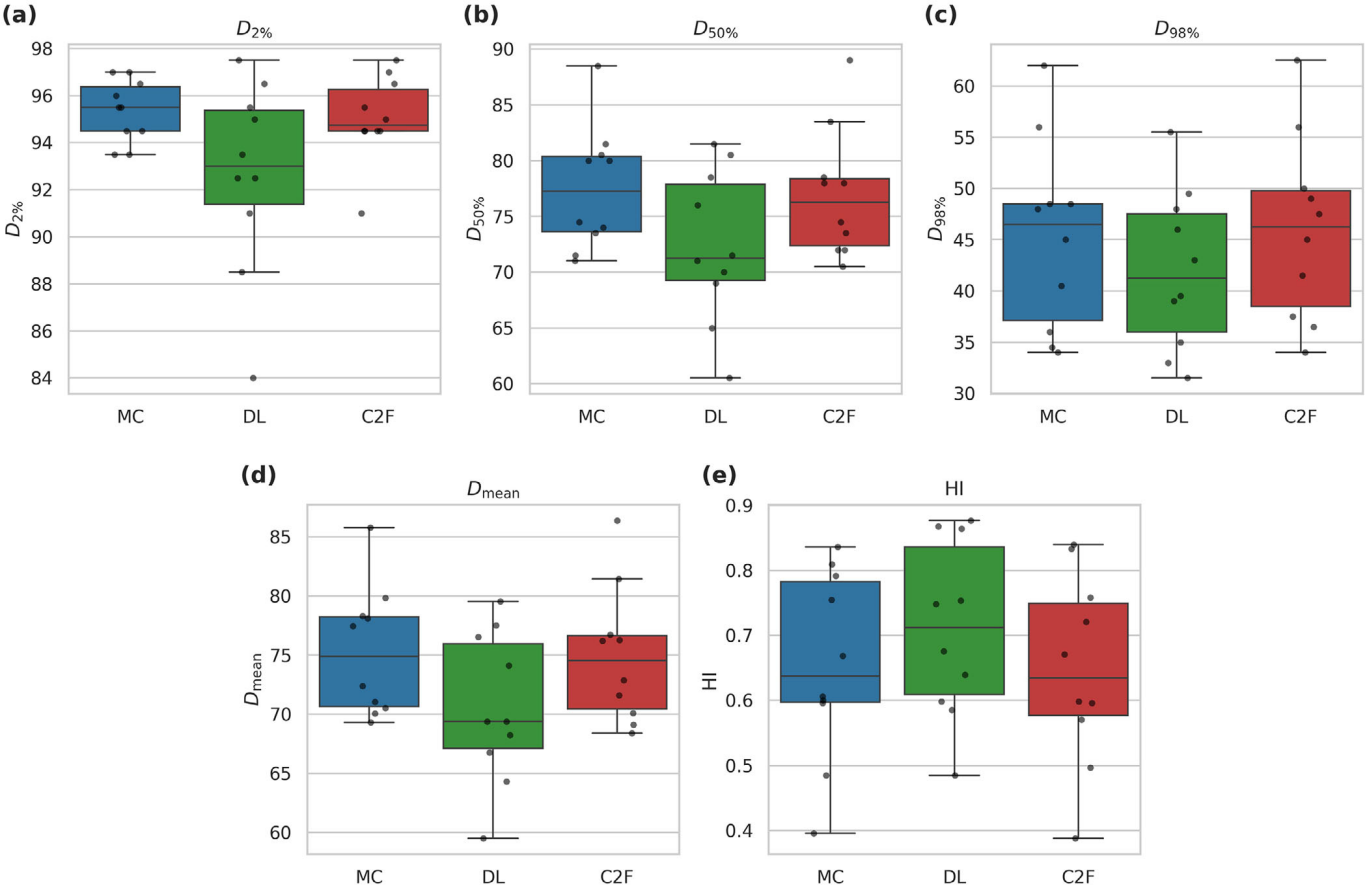
Distributions of plan‐quality metrics across the 10 test patients for the optimization strategies: MC‐only, DL‐only, and coarse‐to‐fine (C2F). (a) near‐maximum dose ($D_{2\%}$), (b) median dose ($D_{50\%}$), (c) near‐minimum dose ($D_{98\%}$), (d) mean dose ($D_{mean}$), and (e) homogeneity index (HI).

**FIGURE 12:**
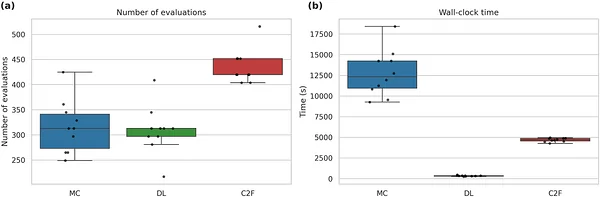
Optimization efficiency across the 10 test patients for the three optimization strategies. (a) Total number of evaluations and (b) total wall‐clock time per patient.

As shown in Figures 10 and 11, the MC‐only and coarse‐to‐fine workflows produced plans of comparable quality, both clearly superior to the DL‐only workflow. Averaged over the test patients, the coarse‐to‐fine and MC‐only plans achieved closely comparable plan quality (mean $D_{mean}$ of 74.9% and 75.3%, mean HI of 0.647 and 0.655), with each producing the better plan in roughly half the patients, whereas the DL‐only plans were consistently worse on the true MC dose (mean $D_{mean}$ 70.5%, mean HI 0.710). These results reflect a trade‐off between the two baselines. The DL‐only workflow is fast, but a small bias in its dose predictor can lead the optimizer to beam parameters that appear optimal according to the predicted dose, yet yield a lower plan quality once the dose is recalculated with MC. The MC‐only workflow does not have this bias, but it runs a full MC simulation at every evaluation, which is computationally expensive. The coarse‐to‐fine workflow combined the strengths of both, matching MC‐only plan quality while accelerating the optimization process by approximately 2.7 ×, reducing the mean optimization time from 12 764 to 4690 s (Figure 12). On average, the coarse‐to‐fine run used 329 evaluations in the coarse stage and 107 in the fine stage (436 evaluations in total). Although this is more evaluations overall, the coarse stage accounted for only 370 s of the runtime, with the 107 fine‐stage MC evaluations accounting for the remaining 4320 s.

**FIGURE 13:**
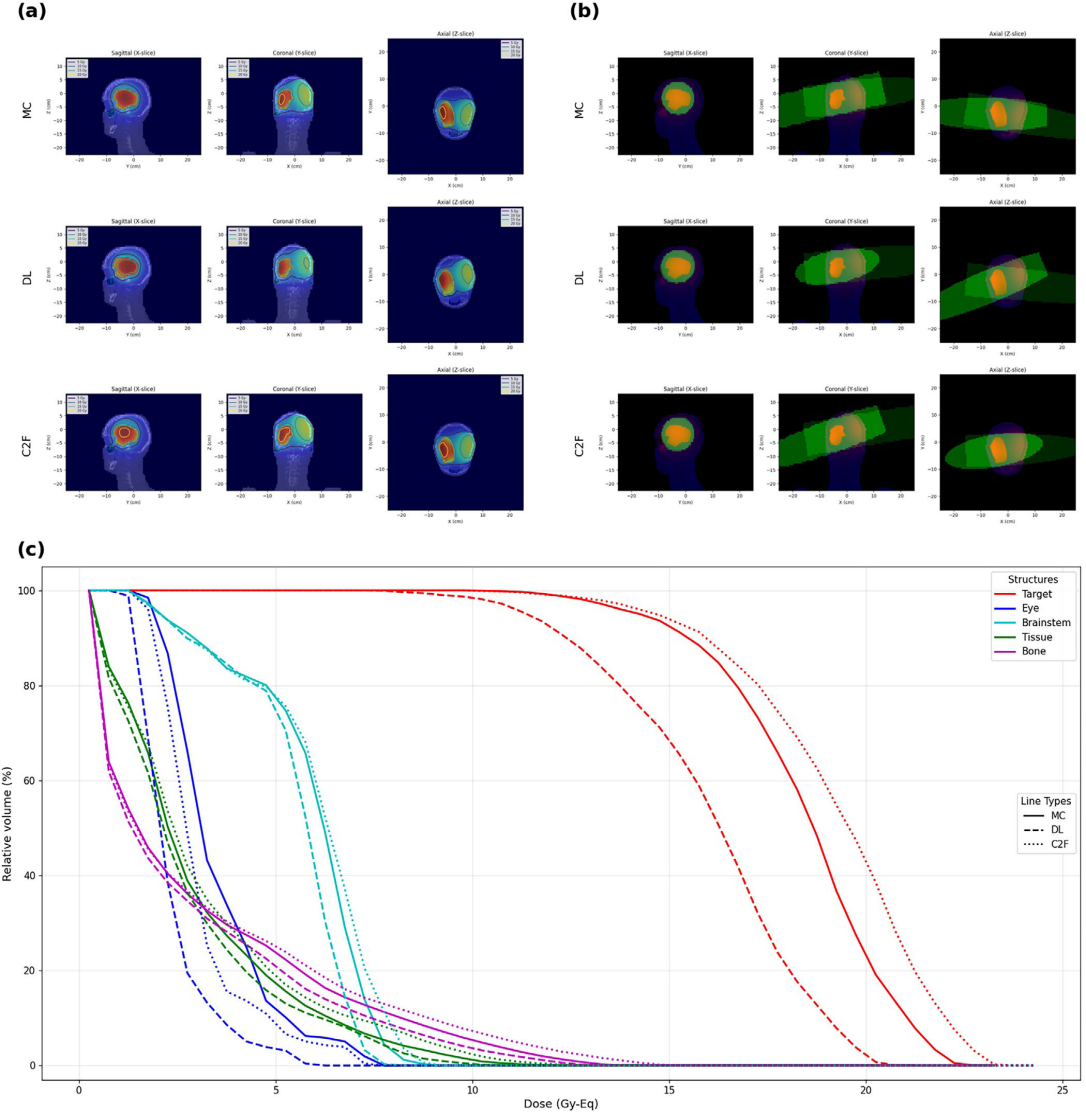
Comparison of (a) 3D dose distributions, (b) beam path projections, and (c) dose‐volume histograms (DVHs) for the MC‐only, DL‐only and coarse‐to‐fine (C2F) optimization strategies, shown for a representative patient.

This result confirms the rationale of the design of our coarse‐to‐fine framework. The DL model rapidly localizes a promising region of the search space in the coarse‐grained stage at negligible evaluation cost, after which the high‐fidelity MC engine refines the solution within a restricted bound in the fine‐grained stage, removing the DL bias. The workflow therefore attains MC‐level plan quality without incurring the full MC computational cost. Figure 13 illustrates this for a representative patient, comparing the 3D dose distributions, beam path projections, and DVHs.

## 4 CONCLUSIONS

In this study, we developed a patient position optimization framework for inverse planning of BNCT that overcomes the limitations of manual forward planning and computationally prohibitive exhaustive searches. By formulating patient positioning as a black‐box optimization problem and solving it using TuRBO, we demonstrated that high quality treatment plans can be generated automatically and efficiently. TuRBO matched the plan quality of grid search while using far fewer dose evaluations, corresponding to a more than fivefold reduction in computation time. By combining a fast DL‐based coarse search with high‐fidelity MC refinement, the coarse‐to‐fine workflow attained plan quality comparable to full MC‐based optimization at substantially lower computational cost.

However, our current approach has certain limitations. Because the DL model was trained on facility‐specific beam data from a previous study, applying the framework at other centers would require retraining the DL model on their own unique beam data. Also, the beam port was also constrained to a fixed 15 cm spherical surface. As the model was trained only at this fixed distance, treating the beam‐port‐to‐skin distance as an optimization variable would likewise require generating a new training dataset and retraining the model. Additionally, the current simulations neglect incident beam divergence. Despite these constraints, our findings suggest that automated inverse planning is a viable pathway for clinical BNCT. Future work will address the physical approximations, particularly incident beam divergence, and will extend the framework in three directions: adding the beam‐port‐to‐skin distance as an optimization variable, analyzing the sensitivity of the objective function weights, and supporting more complex multi‐beam configurations.

## CONFLICT OF INTEREST STATEMENT

The authors declare no conflicts of interest.

## ETHICAL APPROVAL

This study used only publicly available, fully de‐identified human subject data from the GLIS‐RT dataset hosted by The Cancer Imaging Archive (TCIA). No new data were collected from human participants by the authors. Accordingly, institutional review board approval and informed consent were not required for this study.

## Data Availability

The primary imaging dataset (GLIS‐RT) analyzed in this study is publicly available in The Cancer Imaging Archive (TCIA). The simulation data and code generated during the current study are available from the corresponding author upon reasonable request.
